# Thoracic aortic aneurysm in a patient with tuberous sclerosis

**DOI:** 10.1590/1677-5449.160017

**Published:** 2019-05-28

**Authors:** Martin Andreas Geiger, Alex Aparecido Cantador, Ana Terezinha Guillaumon

**Affiliations:** 1 Universidade Estadual de Campinas – UNICAMP, Departamento de Cirurgia, Disciplina de Moléstias Vasculares, Campinas, SP, Brasil.

**Keywords:** thoracic aortic aneurysm, tuberous sclerosis, endovascular treatment

## Abstract

Tuberous sclerosis is a genetic disease with autosomal dominant transmission. Its classic presentation comprises epilepsy, mental deficiencies, and sebaceous adenomas. Aneurysms of the aorta can be detected in people with tuberous sclerosis ranging from children a few months old to young adults. We report the case of a young patient diagnosed with a saccular thoracic aortic aneurysm and tuberous sclerosis who was successfully treated using an endovascular approach.

## INTRODUCTION

Aortic aneurysms are rare among children and young adults. When seen, they tend to be associated with connective tissue diseases such as Marfan and Ehler-Danlos syndromes, vasculitis, or external causes such as traumas.^1^ Case reports have associated aortic aneurysms with tuberous sclerosis (TS) since 1966.^2^

Tuberous sclerosis was first described by the French neurologist Desiré-Magoire Bourneville in 1880. It has autosomal dominant inheritance, but currently 2/3 of cases are sporadic. The disease has a classical triad comprising epilepsy, mental deficiency, and sebaceous adenoma, but it can manifest with multisystemic characteristics, involving the brain, heart, skin, eyes, kidneys, liver, and lung.^2^ We describe a TS case associated with a thoracic aortic aneurysm.

## CASE DESCRIPTION

The patient was a 26-year-old female who was referred for evaluation of a saccular aneurysm of the thoracic aorta and was in continuous follow-up with a neurologist to treat epilepsy with onset at 3 months of age. During investigation she was diagnosed as having TS. She occasionally suffered generalized tonic-clonic crises and was taking anticonvulsants regularly. When bedridden, she adopted the fetal position, with periods of agitation. She had diffuse subcutaneous nodules and physical examination was difficult because of her degree of agitation. Cranial tomography showed focal hypodense lesions in the cortex, subependymal calcifications, and radial banding in the left hemisphere, compatible with TS. Magnetic resonance of the head revealed subependymal astrocytoma of the lateral ventricles. Echocardiogram findings were normal.

**Figure 1:**
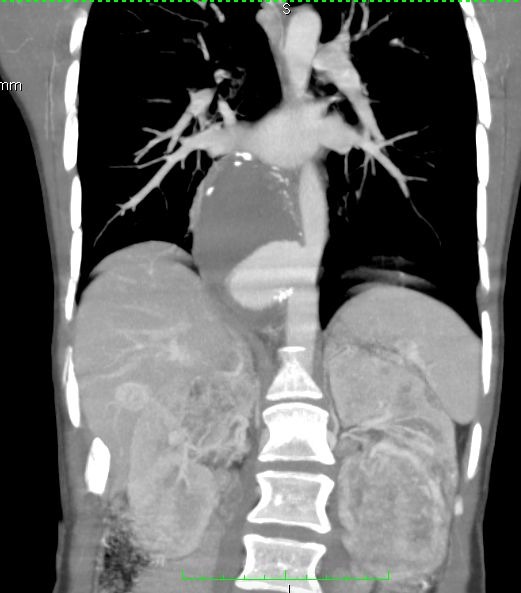
Image of the thoracic saccular aneurysm prior to surgery.

Computed angiotomography of the thoracic and abdominal aorta was ordered, showing a saccular aneurysm of the thoracic descending aorta, with dimensions of 83x53x49 mm (superior-inferior x lateral-lateral x antero-posterior) and a 24 mm neck to the celiac trunk, and also identifying renal angiomyolipomas (Figure 1).

**Figure 2:**
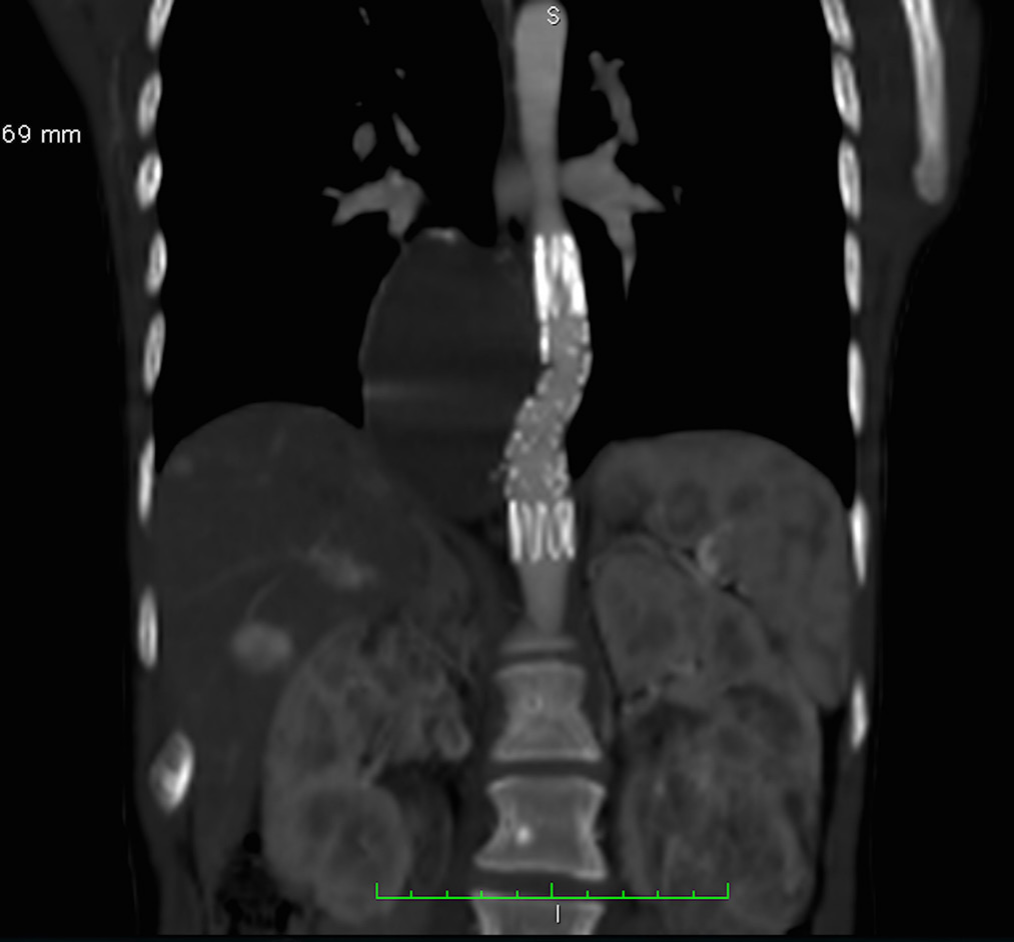
Postoperative follow-up image showing the (patent) prosthesis in place.

The patient underwent surgical treatment, with deployment of a 13x74x20 mm Zenith endoprosthesis (Cook Medical, Bloomington, IN, USA). Control arteriography did not detect any endoleaks. The patient received postoperative care in the ICU for 2 days and was discharged from hospital on the third day after the operation. She was free from complications in outpatients follow-up. Computed angiotomography of the abdominal and thoracic aorta at 6 months after the operation showed the thoracic endoprosthesis patent, with no signs of endoleaks, and the aneurysm sac diameter in regression (Figure 2).

## DISCUSSION

Aortic aneurysms in patients with TS have been described in the literature since 1966.^2^ Onset varies from a few months of life to early adulthood, although one case has been described in a 41-year-old patient.^2^ Josh et al. described 15 cases of aortic aneurysm in patients with TS, 12 of whom had abdominal aortic aneurysms, two who had thoracic aortic aneurysms, and one who had both.^2^ Mean age at diagnosis was 11.7 years. The aneurysms in this sample had large dimensions and a high rate of rupture: occurring in 6 out 15 patients. In a more recent study, Salerno et al. reviewed 21 cases in the literature, the majority of which (17 cases) were diagnosed with aortic aneurysms before 5 years of age. They observed 29% mortality associated with rupture of these aortic aneurysms.^3^

The physiopathogenesis of aortic aneurysms in TS patients is unknown, but it has been observed that there is an abnormality of connective tissue formation, with loss of elastic fibers, similar to that seen in cases of Marfan Syndrome, and build up of mucopolysaccharides, with no inflammatory component.^1^^-^ 4 Moon at al. reported the case of an 8-month old child diagnosed with abdominal aortic aneurysm in which histology also revealed this abnormality.^5^ Kimura at al. also identified loss of elastic fibers with destruction and thinning of the aorta wall.^6^

Since the advent of endovascular techniques, morbidity and mortality associated with treatment of aortic aneurysms related to TS have been reducing progressively.

In the clinical case described, the patient’s pathology was diagnosed when she was 26 years old, but it is believed that, in line with what is described in the literature, she had had the aortic aneurysm since childhood. Endovascular treatment was chosen because of the reduced morbidity and mortality of the technique compared to open surgery.

Other arterial diseases, such as stenosis of medium and large vessels and coarctation of the abdominal aorta, are also described in patients with TS. The low incidence of TS-related aortic aneurysms means that diagnosis is very often made when rupture is imminent. Difficulties with physical examination can also contribute to delayed diagnosis. It is therefore necessary to be aware of the association between these two diseases, to seek for signs and symptoms, and conduct screening tests, abdominal ultrasonography or tomography, to make early diagnosis.^1^^,^ 7 Salerno et al. suggest using duplex ultrasonography for assessment of small children, because it is easily tolerated, does not use contrast, and can be completed quickly; however, this examination is very often inadequate for thoracic examinations.^3^^,^ 5

Since the risk of rupture is elevated, it is suggested that surgical repair be performed at the time of diagnosis.^1^^,^ 3 ^,^ 7 ^,^ 8 Adequate and continuous follow-up with imaging exams is necessary because formation of additional aneurysms is possible.
