# Supplementary material for: Thoracic aortic aneurysm in a patient with tuberous sclerosis
Source: J Vasc Bras. 2019 May 28;18:e20160017. doi: 10.1590/1677-5449.160017 (PMC6634939; doi:10.1590/1677-5449.160017)
Supplement: Translation

# Translation: Aneurisma de aorta torácica em paciente com esclerose tuberosa

**Authors:** Martin Andreas Geiger, Alex Aparecido Cantador, Ana Terezinha Guillaumon

**Affiliations:** 1 Universidade Estadual de Campinas – UNICAMP, Departamento de Cirurgia, Disciplina de Moléstias Vasculares, Campinas, SP, Brasil.

**Keywords:** aneurisma de aorta torácico, esclerose tuberosa, tratamento endovascular

## Abstract

A esclerose tuberosa é uma doença genética de transmissão autossômica dominante. Caracteriza-se, na sua apresentação clássica, por epilepsia, deficiência mental e adenomas sebáceos. Aneurismas de aorta podem acometer desde crianças com poucos meses de vida até adultos jovens portadores de esclerose tuberosa. Apresentamos o caso de uma paciente jovem com diagnóstico de aneurisma sacular de aorta torácica e esclerose tuberosa tratada com sucesso por via endovascular.

## INTRODUÇÃO

Aneurismas de aorta são raros em crianças e adultos jovens. Quando identificados, costumam estar associados a doenças do tecido conjuntivo como as síndromes de Marfan e de Ehler-Danlos, vasculites ou acometimentos externos como trauma^1^. Relatos de casos associam aneurisma de aorta à esclerose tuberosa (ET) desde 1966^2^.

A ET foi descrita inicialmente pelo neurologista francês Desiré-Magoire Bourneville em 1880. Tem herança autossômica dominante; porém, atualmente 2/3 dos casos são esporádicos. É uma doença cuja tríade clássica caracteriza-se por epilepsia, deficiência mental e adenoma sebáceo, mas que pode assumir um caráter multissistêmico, acometendo cérebro, coração, pele, olhos, rins, fígado e pulmão^2^. Relatamos um caso de ET associada a aneurisma de aorta torácica.

## DESCRIÇÃO DO CASO

Paciente de 26 anos, sexo feminino, encaminhada para avaliação por aneurisma sacular de aorta torácica, encontrava-se em seguimento contínuo com neurologista por quadro de epilepsia iniciado aos 3 meses de idade. Em investigação, foi diagnosticada como portadora de ET. Apresentava ocasionalmente crises tônico-clônicas generalizadas e fazia uso de anticonvulsivantes regularmente. Acamada, mantinha posição fetal com períodos de agitação. Apresentava nódulos subcutâneos de acometimento difuso e o exame era dificultado pela agitação da paciente. À tomografia de crânio, apresentava lesões hipodensas focais no córtex, calcificações subependimárias e banda radial no hemisfério esquerdo, compatíveis com ET. Ressonância magnética de crânio evidenciava astrocitoma subependimário nos ventrículos laterais. Ecocardiograma não revelou alterações.

Foi solicitada angiotomografia computadorizada de aorta torácica e abdominal, que evidenciou um aneurisma sacular de aorta torácica descendente, com dimensões de 83x53x49 mm (craniocaudal x laterolateral x anteroposterior), apresentando colo de 24 mm até o tronco celíaco, sendo também identificados angiomiolipomas renais (Figura 1).

**Figura 1:** Imagem do aneurisma sacular torácico prévio à cirurgia.

A paciente foi submetida a tratamento cirúrgico, com implante de endoprótese Zenith (Cook Medical, Bloomington, IN, USA) 13x74x20 mm. Em arteriografia de controle, não foi identificada endofuga. Foi realizado pós-operatório em UTI durante 2 dias, com alta hospitalar no terceiro dia de pós-operatório. A paciente não apresentou complicações no seguimento ambulatorial. Angiotomografia computarizada de aorta torácica e abdominal realizada no sexto mês de pós-operatório evidenciou endoprótese torácica pérvia, sem sinais de endofuga com diâmetro do saco aneurismático em regressão (Figura 2).

**Figura 2:** Imagem no pós-operatório evidenciando prótese locada, pérvia.

## DISCUSSÃO

Aneurismas de aorta em pacientes com ET vêm sendo descritos na literatura desde 1966^2^. O acometimento varia desde crianças com poucos meses de vida até adultos jovens, sendo entretanto descrito um caso de paciente com 41 anos de idade^2^. Josh et al. descrevem 15 casos de aneurisma de aorta em pacientes com ET, dos quais 12 apresentavam aneurisma de aorta abdominal, dois apresentavam aneurisma de aorta torácica e um apresentava ambos^2^. A idade média ao diagnóstico foi de 11,7 anos. Nessa mesma casuística, observou-se que os aneurismas eram de grandes dimensões e com índice alto de ruptura: 6 dos 15 pacientes. Em um estudo mais recente, Salerno et al. revisaram 21 casos da literatura, a maioria dos quais (17 casos) foi diagnosticada com aneurisma de aorta antes dos 5 anos de idade. Observou-se uma mortalidade de 29% associada à ruptura desses aneurismas de aorta^3^.

A fisiopatogenia dos aneurismas de aorta nos portadores de ET é desconhecida; porém, observou-se que existe uma anormalidade na formação do tecido conjuntivo com perda de fibras elásticas, como nos casos de síndrome de Marfan e de acúmulo de mucopolissacarídeos, sem componente inflamatório^1^^-^ 4. Moon et al. relatam o caso de uma criança de 8 meses diagnosticada com aneurisma aórtico abdominal cuja histologia também revelou tal alteração^5^. Kimura et al. também identificaram perda de fibras elásticas com destruição e afilamento da parede da aorta^6^.

Desde o advento das técnicas endovasculares, a morbidade e a mortalidade associadas ao tratamento dos aneurismas de aorta relacionados a ET vêm diminuído progressivamente.

No caso clínico descrito, a paciente teve a patologia diagnosticada aos 26 anos de idade; no entanto, acredita-se, em conformidade com a literatura, que fosse portadora do aneurisma de aorta desde a infância. Optou-se pelo tratamento endovascular devido à menor morbimortalidade da técnica comparativamente à cirurgia aberta.

Outras doenças arteriais, como estenose de médios e grandes vasos, bem como coarctação da aorta abdominal, também são descritas em pacientes com ET. Devido à baixa incidência, o diagnóstico de aneurisma aórtico em portadores com ET muitas vezes acontece na iminência de ruptura. A dificuldade de exame físico pode contribuir para o retardo do diagnóstico. Assim, torna-se necessário o conhecimento da associação entre as duas doenças, a busca por sinais e sintomas e a realização de exames de triagem, ultrassonografia ou tomografia de abdome, para o diagnóstico precoce^1^^,^ 7. Salerno et al. sugerem o uso de ultrassonografia dúplex como método de avaliação em crianças pequenas, por ser facilmente tolerada, não utilizar contraste e apresentar tempo rápido para sua realização; entretanto, esse exame é muitas vezes inadequado para a avaliação torácica^3^^,^ 5.

Como o risco de ruptura é elevado, sugere-se reparo cirúrgico no momento do diagnóstico^1^^,^ 3 ^,^ 7 ^,^ 8. Seguimento adequado e contínuo, com exames de imagem, torna-se necessário, pela possibilidade de formação de novos aneurismas.
